# Toward a new understanding of the links between poverty and illegal wildlife hunting

**DOI:** 10.1111/cobi.12622

**Published:** 2015-11-23

**Authors:** Rosaleen Duffy, Freya A. V. St John, Bram Büscher, Dan Brockington

**Affiliations:** ^1^ Department of Development Studies, SOASUniversity of LondonThornhaugh Street, Russell SquareLondonWC1H 0XGUnited Kingdom; ^2^ Durrell Institute of Conservation and EcologySchool of University of KentCanterburyKentCT2 7NRUnited Kingdom; ^3^ Sociology of Development and Change GroupWageningen UniversityHollandsweg1 6706KNWageningenThe Netherlands; ^4^ Institute for Development Policy and Management, School of Environment, Education and DevelopmentThe University of ManchesterArthur Lewis Building, Oxford RoadManchesterM13 9PLUnited Kingdom

**Keywords:** ivory, poaching, rhino horn, rural development, wildlife trade, caza furtiva, cuerno de rinoceronte, desarrollo rural, marfil, mercado de fauna

## Abstract

Conservation organizations have increasingly raised concerns about escalating rates of illegal hunting and trade in wildlife. Previous studies have concluded that people hunt illegally because they are financially poor or lack alternative livelihood strategies. However, there has been little attempt to develop a richer understanding of the motivations behind contemporary illegal wildlife hunting. As a first step, we reviewed the academic and policy literatures on poaching and illegal wildlife use and considered the meanings of poverty and the relative importance of structure and individual agency. We placed motivations for illegal wildlife hunting within the context of the complex history of how wildlife laws were initially designed and enforced to indicate how hunting practices by specific communities were criminalized. We also considered the nature of poverty and the reasons for economic deprivation in particular communities to indicate how particular understandings of poverty as material deprivation ultimately shape approaches to illegal wildlife hunting. We found there is a need for a much better understanding of what poverty is and what motivates people to hunt illegally.

## Abstract (es)

Hacia un Nuevo Entendimiento de la Conexión entre la Pobreza y la Caza Ilegal de Animales

**Resumen**

Las organizaciones de conservación han incrementado cada vez más la preocupación con respecto a la caza ilegal y el mercado de fauna. Los estudios previos han concluido que la gente caza ilegalmente porque son financieramente pobres o carecen de estrategias alternativas de sustento. Sin embargo, ha habido pocos intentos por desarrollar un entendimiento más robusto de las motivaciones detrás de la caza ilegal contemporánea. Como primer paso, revisamos la bibliografía académica y política sobre la caza furtiva y el uso ilegal de la fauna y consideramos los significados de la pobreza y la importancia relativa de la voluntad individual y de estructura. Colocamos las motivaciones para la caza ilegal dentro del contexto de la historia compleja de cómo las leyes de vida silvestre estuvieron inicialmente diseñadas e impuestas para indicar cómo las prácticas de caza de comunidades específicas estuvieron criminalizadas. También consideramos la naturaleza de la pobreza y las razones de la privación económica en comunidades particulares para indicar cómo los entendimientos particulares de la pobreza como material de privación eventualmente forman a las estrategias contra la caza ilegal. Encontramos que existe una necesidad de entender de mejor manera qué es la pobreza y qué motiva a las personas a cazar ilegalmente.

## Introduction

Recent increases in illegal wildlife hunting and trading have attracted international attention, particularly of high‐value endangered species vulnerable to illegal international trade. A recent report by the United Nations Environment Programme notes that illegal hunting of different species demand diverse strategies, including demand reduction campaigns, use of anti‐money laundering regulations, development of surveillance networks, and increased use of force (Nellemann et al. 2014). Illegal hunting covers a very wide range of activities associated with complex motivations and drivers that require different responses. It can be driven by demand for high‐value luxury products (e.g., sturgeon [*Huso huso*] hunting to meet international demand for caviar or Chiru hunting [*Pantholops hodgsonii*] in China to produce shatoosh wool). In contrast, certain species of bears are in demand for the production of cheap pharmaceutical products, and antelopes and primates are hunted for meat for subsistence and sale. Through an examination of policy and academic literatures on poaching and illegal wildlife use our goal was to develop a richer understanding of contemporary illegal hunting by connecting theories explaining individual motivations with those focusing on broad social, economic, and political drivers.

Current understanding of illegal hunting is hampered by a lack of data. Moreover, it is frequently approached primarily as a conservation concern rather than an issue of poverty and development. Yet, the argument that it results from poverty is widely used (Adams et al. 2004a *a*; Nellemann et al. 2014). We argue that effective conservation strategies need to move on from this characterization and recognize the complexities of motivations and political‐economic contexts so that illegal wildlife hunting can be addressed in a more effective, socially and environmentally just manner.

We placed illegal wildlife hunting in the context of a wider conceptual debate in the social sciences, that of structure and agency. This allowed us to discuss their relative importance in motivations to engage in illegal hunting and to argue that a broader view of illegal hunting and poverty is needed that places greater emphasis on historical and contemporary political‐economic and social contexts.

## Defining Illegal Wildlife Hunting

We use the term *illegal wildlife hunting* rather than *poaching* in order to be explicit about the type of behavior we are referring to: any hunting or wildlife extraction not explicitly sanctioned by the state or private owners of wildlife. Here context matters, for example, a particular act (shooting a wild animal) might be illegal within a protected area but legal when the animal crosses the park boundaries or threatens people or property. This context includes the colonial history of hunting regulations; laws cannot be uncritically accepted as given (MacKenzie 1988; Jacoby 2003; Neumann 2004). For example, colonial legislation removed hunting rights from Africans to protect the sports hunting and safari industries for European colonizers (MacKenzie 1988; Jacoby 2003; Neumann 2004). These laws criminalized some African livelihood practices and were often refined and extended by states after independence. The origins of illegality in hunting partly explain why some communities in Sub‐Saharan Africa continue to resist legislation to protect wildlife. In essence those communities maintain that they have a right to access and use wildlife (Mackenzie 1988; Carruthers 1995; Garland 2008; Roe 2008b *b*; Robbins et al. 2009; Fischer et al. 2013).

The very nature of illegal wildlife hunting hampers understanding of it. Published research on the motives of illegal hunters is scant largely because few are willing to identify themselves (St. John et al. 2010). The available work tends to distinguish between subsistence and commercial hunting. The former typically targets small game (e.g., antelope), to meet food needs, hunted with simple technology (e.g., traps and snares) and tends to have a minimal impact on wildlife populations (Mackenzie 1988; Bodmer & Lozano 2001; Adams 2004b *b*; Adams 2009; Lowassa et al. 2012; Fischer et al. 2013; Twinamatsiko et al. 2014; Harrison et al. 2015). By contrast, commercial hunters typically operate within organized groups that target commercially valuable species, such as rhinoceroses, elephants, orangutans (*Pongo abelii*, *P. pygmaeus*), and tigers, and use more advanced technologies, including firearms and geographic positioning systems (Ellis 1994; Ellis & Reeve 1995; Leakey 2001; Duffy 2014; Nellemann et al. 2014; Harrison et al. 2015). However, the distinction between subsistence and commercial hunting can be blurred because meat may be hunted to supplement both diets and income (Mackenzie et al. 2011; Vega et al. 2013). Furthermore, illegal wildlife hunting for subsistence purposes can become commercial. For example, subsistence hunting can transform into commercial hunting in response to the arrival of logging companies in remote forests, where a workforce has to be fed or transport links give easier access to urban markets (Nellemann et al. 2014; Harrison et al. 2015). In a similar vein, hunting in conflict zones (discussed below) cannot be easily categorized as subsistence or commercial because it blends elements of both (Redford 1992; Duffy 2010; Nellemann et al. 2014).

## Poverty and Illegal Wildlife Hunting

Poverty is often perceived as the root cause of illegal wildlife hunting because poor people hunt illegally to satisfy basic material needs (Mackenzie et al. 2011; Twinamatsiko et al. 2014; IUCN et al. 2015). For example, a study of Bwindi National Park in Uganda showed that those arrested for unauthorized activities in the national park were significantly poorer and more likely to live closer to the national park and farther from trading centers than others (Twinamatsiko et al. 2014). A recent study on the links between poverty and wildlife crime in Uganda indicated that one of the most effective ways to reduce illegal wildlife hunting is poverty alleviation (Harrison et al. 2015). Similarly, more effective involvement of the rural poor in both development and conservation projects is also advocated (Roe 2008a; Roe (ed) 2013; Sanderson & Redford 2003; Roe 2015; Roe et al. 2015). However, poverty is a complex condition, which makes these claims opaque. What form of poverty and poverty alleviation are referred to? Challender and MacMillan (2014) note, poverty is not a singular category, and they draw attention to the importance of relative poverty in driving illegal wildlife hunting. However, we go a step further to argue that a much more sophisticated analysis of what constitutes poverty, relative poverty, and inequality are needed to develop a better understanding of what the ultimate drivers of illegal wildlife hunting are. We could not review debates on poverty in much detail (but see Hulme 2010), but it is important to explain the relevance of engaging with the meaning of *poverty*.

Within debates about conservation there has been a tendency to rely on largely economic definitions of *poverty* that focus on material deprivation. For example, in a systematic review of evidence of the links between poverty and biodiversity, 70% of published papers that addressed poverty as part of conservation used income as the key measure (Roe et al. 2014). It follows logically that illegal wildlife hunting can supposedly be tackled via provision of paid employment (e.g., as rangers and tour guides), which increases levels of material wealth, or alternative income generation or disbursement schemes, such as development of markets for local agricultural produce or funds from selling safari hunts and photographic tourism (Barrett & Arcese 1995; Adams & Infield 2002; Roe et al. 2010; Spenceley & Meyer 2012). Based on this logic, MacKenzie et al. (2011) suggest that because illegal resource use in Kibale National Park, Uganda, is not driven by food insecurity, then the approach of enforcing exclusion of local communities is justified.

The argument that people hunt illegally because they are materially poor is repeated in powerful policy arenas. For example, the International Conservation Caucus Foundation (ICCF), which involves one‐third of the membership of the U.S. Congress, has stated that extreme poverty in Africa is the source of illegal wildlife hunting as well as radicalization (ICCF 2014). The same view was expressed in the high‐level meeting on illegal wildlife trade hosted by the U.K. Government in May 2013 (Government of the United Kingdom 2013). We do not dispute the argument that material deprivation matters, but there are 3 problems with defining poverty only in material terms. First, the understanding of poverty is itself impoverished; it does not capture what being poor means. Following Sen's (1999) formulation, poverty also encompasses a lack of power, prestige, voice, and an inability to define one's future and day‐to‐day activities, which are difficult to measure in quantifiable terms. Second, it denies that the poor have agency and are able to lead fulfilling and meaningful lives. Accordingly, illegal wildlife hunting may not simply be a way of averting want and deprivation, it may be a means of seeking and affirming identity, status, lifeways, custom, and local prestige. A current challenge for conservation is how to measure human well‐being (Milner‐Gulland et al. 2014) and multidimensional poverty in order to capture factors such as voice, prestige, and status (Sen 1999). It is critical to develop an understanding of how such factors relate to behaviors, such as illegal wildlife hunting, so that they can be integrated into conservation measures.

MacDonald's (2005) work on the Himalayan ibex trophy hunting scheme in northern Pakistan underlines the importance of this perspective (2005). Aiming to conserve a declining ibex population, the IUCN worked to establish a trophy‐hunting scheme that required local people to refrain from hunting, an activity they had engaged in for centuries and sold hunting permits to international hunters. The proceeds from the sale of permits were split between local people and the national government. However, the scheme failed to halt local ibex hunting, largely because hunting was a source of prestige and status. Distributing money from trophy hunts created new sources of prestige. It did not remove all the incentives to hunt because those controlling the money were different from those who previously controlled the meat (also see Marks 2001).

Third, defining poverty purely in economic terms allows it to be presented as a technical issue that can be solved through the provision of alternative sources of income. This in turn lends itself to technical interventions that are limited to economic incentives (e.g., alternative livelihood schemes) and disincentives (e.g., enforcement to make wildlife hunting illegal). But a broader approach to understanding poverty (such as Sen's analysis), would see interventions aimed at reducing illegal wildlife hunting embedded or mainstreamed into wider development initiatives that address the factors that drive the development of illegal wildlife hunting in the first place (IUCN et al. 2015).

## Structure, Agency, and Illegal Wildlife Hunting

We suggest recasting the debate with a foundational approach in social sciences—that of structure and agency. Those who debate structure and agency discuss the extent to which decisions made by individual agents are constrained by the structural context in which they exist (Hay 2002). A more thorough and robust engagement with this key debate would allow for a fuller understanding of why people hunt illegally.

### Structural Approaches to Illegal Wildlife Hunting

Structural explanations emphasize the importance of the wider context, which recognizes the complex role of international demand. Although material poverty may encourage people to hunt illegally, such poverty can only become a driver if there is demand from wealthier communities (IFAW 2008; Duffy 2010; Ayling 2013; Challender & Macmillan 2014). For example, a report by TRAFFIC‐ASIA examined the drivers of the illegal wildlife trade and concluded that the increase in illegal trading of wildlife was directly related to the rise in incomes in Southeast Asia. This report added an oft‐missed aspect, namely the complexity of the networks involved and the wide range of cultural, political, economic, and social contexts in which these function. It linked local‐level rural harvesters, professional hunters, traders, wholesalers and retailers with the final consumers of wildlife in locations distant from the source of the product. The illegal wildlife trade provided varying forms of economic support to different parts of the network: a source of regular income, a safety net, or as profitable business (TRAFFIC 2008).

The importance of structural contexts are also discernible in the ways that illegal wildlife hunting has been used as a financial underpinning for conflicts across Sub‐Saharan Africa for some time, including Uganda in the 1970s and 1980s Angola and Mozambique in the 1980s, and the Great Lakes region since 1996 (Ellis 1994; Humphreys & Smith 2011, 2014). The rise in illegal hunting in Central African Republic (CAR) and its relationships to regional security issues (Chad, Cameroon, CAR, and Gabon) was detailed in a report by UN Secretary General, Ban Ki Moon (UN 2013). Zakouma National Park in Chad has also suffered hunting by rebel groups to fund cross‐border wars. Garamba National Park in Democratic Republic of Congo was used as a base by the Lord's Resistance Army (LRA) in 2012, and the LRA financed operations through the illegal hunting and sale of elephant ivory (UN 2013; UNEP et al. 2013; White 2014). However, these characterizations have been questioned. Lombard suggests that linking illegal ivory hunting to LRA and Janjaweed relies on simplistic labels that are strategically used by governments in the region to entice external help or to demonize illegal hunters as outsiders from neighboring states (Lombard 2012; White 2014). Furthermore, conflicts can produce large‐scale population displacements and refugee camps; refugees and internally displaced people can turn to illegal wildlife hunting to feed themselves or to earn cash income. A study by TRAFFIC East/Southern Africa found that rations provided in refugee camps in Tanzania were not sufficient, so refugees turned to hunting to meet protein needs (Jambiya et al. 2007).

Hunting that is linked to the illegal international wildlife trade is also portrayed as part of wider concerns about national and global security risks. For example, the chief executive officer and chairman of Conservation International, recently called poverty, extinction and radicalism a “perfect storm” that threatens global stability (Conservation International 2013). Similarly, the Wildlife Conservation Society has launched its 96 Elephants campaign, which has 3 central pillars Humans and Elephants, Terror and Ivory, and Heroes and Hope that link poverty, regional instability, illegal wildlife hunting, and terrorism (Wildlife Conservation Society 2014). Furthermore, some conservation nongovernmental organizations (NGOs) and national governments have tried recently to claim that illegal wildlife hunting for ivory in the Horn of Africa is being used to fund Al Shabaab (a militia group based in Somalia but allied to Al Qaeda since 2012). These claims were reported in a 2012 report by Elephant Action League (EAL) (Kalron & Crosta 2012) and were taken up in the international media and in policy discussions (Lawson & Vines 2014; White 2014). Elsewhere, we questioned the evidence base of these claims (the EAL report was based on very limited empirical research in Somalia) and contend that the blunt links made between illegal hunting for ivory and terrorism are overly simplistic (Duffy et al. 2015). A recent report from UN United Nations Environment Programme (UNEP) and INTERPOL note that claims Al Shabaab was trafficking 30.6 t of ivory per annum (representing 3600 elephants per year) through southern Somalia are “highly unreliable” and that the main sources of income remain charcoal trading and ex‐pat finance (Nellemann et al. 2014).

Furthermore, in making the link to global security, the underlying reasons for the appearance and activities of militia and rebel groups are left as a black box and are not discussed. It means that complex social, economic, and political issues are largely framed as a question around the impact of illegal wildlife hunting on wildlife rather than approaching them in more expansive and politically sensitive ways that help us understand why people hunt illegally in the first place. Brashares et al. (2014) partially meet this need when they argue that policies to tackle illegal wildlife hunting and trafficking need to look at underlying causes, primarily by strengthening local tenure over resources. This is important, but focusing solely on tenure overlooks important factors. One needs to examine how social, economic, and political inequalities are produced in the first place, how they might encourage individuals or communities to engage in illegal wildlife hunting, and then tackle those, which is a much larger task.

The significance of approaches that emphasize the importance of structural context, indicate how context constrains individual choice. Once this is understood, one may be able to design better policy responses. These could include tackling the historical removal of hunting rights, dispossession from land to create protected areas, creation of better education and employment opportunities in urban areas, and more rural‐development efforts. Put simply, we contend that structural approaches are needed to shape and define possible policy responses to tackle illegal wildlife hunting and that these will look very different from current approaches.

### Agential Approaches to Illegal Wildlife Hunting

The second set of explanations for illegal wildlife hunting draws on approaches that emphasize agency. Such explanations acknowledge the influence of the wider social context but place emphasis on the agency of individuals. Ultimately agential approaches are anchored in the idea that people have the capacity to act independently (Ostrom 2010; St. John et al. 2013) and revolve around understanding how individuals respond to the circumstances they face; these approaches are anchored in disciplines such as economics and social psychology.

Economic analyses of human behavior are traditionally underpinned by a model of rational choice. In such analyses, rational actors aim to maximize their utility or “level of satisfaction” (Janssen et al. 2010; Ostrom 2010). Models of rational choice have made an important contribution to understanding how people involved in illegal wildlife hunting may respond to enforcement activities such as ranger patrols. For example, Milner‐Gulland and Leader‐Williams (1992) showed that the probability of detection significantly influences decisions to hunt rhinoceros and elephants illegally in the Luangwa Valley, Zambia. Their models also suggest that local people engage in illegal hunting and organized gangs react differently to law enforcement. Integrated conservation and development projects (ICDPs) may induce the former to refrain from hunting illegally, the latter could only be deterred through increased enforcement.

Models of rational decision makers have also been used to explore decisions made by households that undertake multiple livelihood activities (Milner‐Gulland 2011). These household utility models are comparable to those used in agricultural and development economics and have been used to explore how individual households allocate labor to different livelihood activities (Milner‐Gulland 2012; also see Keane et al. 2008). A modeling study by Damania et al. (2005) shows how ICDPs could have unintended consequences. Their model provided a premium price for crops in order to raise the opportunity cost of hunting, a common suggestion for reducing legal and illegal wildlife hunting. Counterintuitively, their results indicate that although the proportion of labor devoted to agriculture increases, the extra income is invested in increased bushmeat consumption and new hunting gear, which would perversely enable hunter‐farmers to target more vulnerable and commercially valuable species. The overall impact on reducing hunting (legal and illegal) was ambiguous (Damania et al. 2005; Milner‐Gulland 2012).

The decisions individuals make are also influenced by their preference for present over future benefits. With respect to poor people living in rural areas of the global South, these dynamics are poorly understood. For example, it is assumed that the poor are preoccupied with difficult livelihoods in the present and as a result have high discount rates that result in overexploitation of local resources to fulfill immediate needs. However, evidence from food security analysts indicates that the poor often eat less to preserve productive capital and chances of producing food in the future. This calls into question the commonly held view of poverty‐induced environmental degradation (Moseley 2001).

While still assuming that individuals make rational decisions about how to act based on an evaluation of information available to them, social psychologists use different types of predictors from economists. Social psychologists highlight interactions of internal (e.g., attitudes and values) and external (e.g., other people and availability of resources) influences on behavior by measuring these cognitive components (Manfredo et al. 2009; also see Litchfield 2013). One influential approach is the theory of planned behavior (Ajzen 1991). This theory states that a person's behavior can be predicted by 3 factors: attitude, subjective norms (perceived social pressure), and perceived degree of control over performing a behavior (e.g., availability of required knowledge, skills, and resources). The relative importance these factors have on people's behavior differs from one behavior to another. For example, Williams et al. (2012) show that training could encourage people to cultivate xaté (*Chamaedorea ernesti‐augusti*) in Belize rather over harvest wild plants. The training focused on increasing technical knowledge and developing a perception that individuals could succeed in cultivating it; attitudes and norms barely influenced this behavior. In contrast, attitudes and norms were both important predictors of landowners’ intentions to conserve forest in the agricultural frontier of South American Gran Chaco (Mastrangelo et al. 2013; also see a study on illegal killing of jaguars [*Panthera onca*] by ranchers [Marchini & Macdonald 2012]). Identifying which factors most strongly relate to people's intention to engage in behaviors of conservation concern (e.g., illegal wildlife hunting) can provide valuable insights when one seeks to influence behavior.

The concept of individual agency is particularly pertinent to the discussion of illegal wildlife hunting. Based on logical agential approaches, positive incentives (e.g., receipt of benefits) encourage compliance, whereas negative incentives (e.g., risk of punishment) deter rule breaking. This notion is exemplified in discussions on the illegal hunting of elephant and rhinoceros because many arguments, both for and against legalized trade, are based on the premise that individuals are incentivized by the promise of rewards (Biggs 2013a, 2013b; Duffy 2013).

## Policy Implications

Debates about the structural and agential explanations for illegal wildlife hunting matter; they are not simply theoretical debates. They shape and inform conservation strategies to address the problem on the ground, for better or worse.

Many conservationists are aware of the complex interlinkages between wider contexts and individual motivations. Confronted with complex and pressing challenges surrounding illegal wildlife hunting, those responsible for protected area management, for example, may implement approaches that tackle agential factors as a short‐term or immediate response (e.g., through capacity building projects and community engagement projects, see Baker et al. 2012) while working toward addressing structural or contextual factors in the longer term. However, their ability to produce the scale of structural change needed to develop socially just forms of conservation is limited. Specific conservation projects (on their own) cannot overturn the social, economic, and political factors that produce illegal wildlife hunting in the first place; such fundamental change requires much broader global scale shifts.

It is precisely because of the need to work at scale that we should be aware of what sorts of explanation, and diagnoses of the problem, are employed. Explanations that emphasize the importance of structural context question the effectiveness of conservation interventions that are built solely on the premise that either positive or negavie incentives targeting individual behavior will lead to effective, sustainable solutions for biodiversity conservation. Management of natural resources is widely dependent on negative incentives and on rules prohibiting certain activities. Wider questions of social justice (that go beyond the immediate concerns of economic deprivation) are not adequately addressed. However, enforcement is financially costly, and provision of adequate security prohibitively expensive for many states (Jachmann & Billiouw 1997).

Critics of approaches to illegal wildlife hunting that rely on the use of force and local community exclusion from protected areas argue that it is important to address the broader factors that produces illegal wildlife hunting in the first place (Brockington et al. 2008; Büscher 2013). Reliance on deterrence creates poor relations between conservation authorities and local people by restricting access to resources that may have an important (or irreplaceable) role (Infield 2001). Stern (2008) argues that trust and legitimacy between protected‐area staff and local people are key factors for voluntary compliance, where general agreement with formal regulations does not necessarily exist.

Higher rates of illegal hunting in Africa are increasingly used to justify the use of force, including greater use of arms, shoot‐to‐kill policies, expansion of ranger numbers, contracting of security services to the private sector, and use of new technologies (drones, camera traps, thermal imaging) (Duffy 2014; White 2014; Sandbrook 2015). For instance, UN Secretary General Ban Ki Moon claims that a more militarized approach to conservation law enforcement is needed (UN 2013). As we argue elsewhere, greater levels of enforcement by states, NGOs, or private‐sector operators may produce quick fixes in the short term, but they are problematic (Duffy et al. 2015). In the Liwonde National park in Malawi, South African private military company personnel were used to train the park rangers. Later, park employees were implicated in over 300 deaths, 325 disappearances, 250 rapes, and numerous instances of torture from 1998 to 2000 in this park alone (Neumann 2004). Such forceful approaches can be counter‐productive and can alienate local communities (Peluso 1993; Neumann 2004; Dressler et al. 2010; Lunstrum 2013).

Agential explanations for illegal wildlife hunting are also discernible in the strategies that rely on systems of positive incentives designed to encourage people to comply with wildlife regulations. Positive incentives assume that if money or benefits in kind are given to communities or individuals they will be encouraged to behave in a certain way (e.g., refrain from illegal resource extraction). Since the 1980s, efforts to integrate local people into conservation efforts have gained wide support (e.g., ICDPs and community based natural resource management). Strategies for achieving participation have usually focused on economic links between people in communities and protected areas. Most typically, this relates to the potential income to be made from alternative livelihood strategies, including safari tourism, trophy hunting, and sale of products (Barrett & Arcese 1995; Roe et al. 2015).

The focus on economic incentives assumes that market forces will protect the environment. The arguments deployed in favor of incentives as a means of reducing illegal wildlife hunting are apparent in claims that tourism can reduce poverty, provide economic incentives to individuals and communities, and encourage people to change behaviors towards wildlife (UNEP et al. 2013). However, despite a range of initiatives and considerable donor investment it has proved difficult to provide tangible benefits from conservation to local communities, especially in Sub‐Saharan Africa. This can be because most protected areas do not create sufficient revenue to off‐set the costs to communities of maintaining them (Emerton 1998; Newmark & Hough 2000; Dressler et al. 2010) or because the distribution of benefits is uneven (Bandyopadhyay & Tembo 2010; Richardson et al. 2012). In addition, other studies indicate that provision of alternative livelihoods simply means they become additional rather than alternative sources of income; therefore, although household well‐being may increase, illegal wildlife hunting may continue (Ferraro & Kramer 1997; Ostrom 2010).

Explanations that emphasize the importance of structural context call into question the effectiveness of such incentive schemes because they do not tackle the historical, economic, social, and political factors that produce illegal wildlife hunting in the first place. These factors could include problems arising from eviction and displacement from protected areas. Addressing these factors means conservationists should design approaches that enhance wider forms of rural development to give local communities a genuine range of choices (or freedoms in Sen's [1999] terms) to shape their own lives or at a minimum that engage local communities as managers of and participants in wildlife conservation schemes (Roe et al. 2015). More broadly, policies to tackle illegal wildlife hunting would need to be embedded within and linked to policies that promote just social, political, and economic relations. The danger of policies promoting greater enforcement alone is that even if they could succeed in creating small islands of relative peace inside protected areas, they would not do anything and may in fact exacerbate the issues found beyond them, which ultimately cause illegal wildlife hunting in the first place.

## Conclusion

Our exploration of the literature on the links between poverty and illegal wildlife hunting reveals that understanding is limited and conservationists need to take a more expansive view of what constitutes illegal wildlife hunting, what motivates people to hunt illegally, and how to tackle the problem. Rather than simply seeing hunting as a matter of legality or illegality, we suggest hunting needs to be understood in its historical, social, and political context (MacKenzie 1988). This means acknowledging that some communities could regard laws that criminalize their continued use of wildlife as unjust precisely because these laws were instituted by colonial regimes or post‐independence states that communities may regard as oppressive rather than representative. The Kasane Statement on Illegal Wildlife Trade (March 2015) acknowledges the importance of community engagement much more fully than the earlier London Declaration, which may allow for space to debate more nuanced approaches.

Taking a more expansive view of what illegal wildlife hunting is helps one critically assess the ways that links are being developed between poverty, hunting, and security. It is clear that claims that illegal wildlife hunting is driven by poverty (defined as material deprivation) which leads to radicalization, and helps fund conflicts and terrorism are based on limited information, but they have been taken up with remarkable speed by a wide range of organizations (including the U.S. government); the acceptance of such arguments relies on the simplistic idea that poverty leads to radicalism and that illegal wildlife hunting offers a revenue stream. This assumption has the potential to persuade conservationists to engage in counter‐productive and forceful responses to illegal wildlife hunting (Duffy et al. 2015). A further risk is that in some areas as enforcement intensifies, illegal wildlife hunting will shift to hunting of less well‐protected wildlife populations (Roe et al. 2015). More sophisticated and nuanced understanding of these dynamics needs to be developed.

To do so, we suggest conservation researchers engage more fully with the social sciences, especially regarding the meanings of *poverty* and the relative importance of structure and agency (Sandbrook et al. 2013). This will help reveal the underlying assumptions in debates about the links between poverty and illegal wildlife hunting and set the stage for a fresh approach. Therefore, we suggest 2 next steps for researchers and conservationists alike. First, conduct, with a variety of methods, more research into the links between poverty and illegal wildlife hunting and use novel methods to investigate sensitive topics (Nuno & St. John 2014) and conduct qualitative and ethnographic work with illegal hunters themselves to gain a better understanding of motivations. This research should be accompanied by greater self‐reflection by researchers on how the initial framing of the issue of illegal wildlife hunting helps shape and determine the kinds of information produced. Second, based on an improved understanding of illegal wildlife hunting, devise responses to illegal wildlife hunting that address social inequalities and are sensitive to historical, social, political, and economic contexts. Effective responses will require viewing illegal wildlife hunting as a challenge related to development rather than purely conservation.
